# Contributing Factors of Uneven Climatic Aging for Polymeric Composite Materials: Methods and Modelling

**DOI:** 10.3390/polym15061458

**Published:** 2023-03-15

**Authors:** Mikhail P. Lebedev, Oleg V. Startsev, Anatoly K. Kychkin, Mark G. Petrov, Michail M. Kopyrin

**Affiliations:** 1 Siberian Branch of the Russian Academy of Sciences Federal Research Center ”Yakut Scientific Center SB RAS”, 2 Petrovskogo str., 677000 Yakutsk, Russia; 2 Siberian Branch of the Russian Academy of Sciences V.P. Larionov Institute of Physical and Technical Problems of the North, 1 Oktyabrskaya str., 677000 Yakutsk, Russia; 3 Federal State Unitary Enterprise “S.A. Chaplygin Siberian Aeronautical Research Institute”, 21/1 Polzunova str., 630051 Novosibirsk, Russia

**Keywords:** polymer composite materials, aging, internal stresses, thermo-moist cycling, microcracks, strength gradients

## Abstract

Regarding a wide variety of PCMs, the materials’ strength properties which decrease no more than 20% after 30 years of operation are of special interest. One of the important regularities of the climatic aging of PCMs is the formation of gradients of mechanical parameters across the thickness of the plates. The occurrence of gradients must be taken into account when modeling the strength of PCMs for long periods of operation. At present, there is no scientific basis for the reliable prediction of the physical-mechanical characteristics of PCMs for a long period of operation in the world of science. Nevertheless, “climatic qualification” has been a universally recognized practice of substantiating the safe operation of PCMs for various branches of mechanical engineering. In this review, the influence of solar radiation, temperature, and moisture according to gradients of mechanical parameters across the thickness of the PCMs are analyzed according to the data of dynamic mechanical analysis, linear dilatometry, profilometry, acoustic emission, and other methods. In addition, the mechanisms of uneven climatic PCM aging are revealed. Finally, the problems of theoretical modeling of uneven climatic aging of composites are identified.

## 1. Introduction

Polymeric composite materials (PCMs) based on glass, carbon, basalt, organic, and other fibers are widely used in various branches of mechanical engineering [1,2], and their strength characteristics decrease over time due to aging [3,4,5]. Designing engineering elements always requires information on the durability of used PCMs in conditions of exposure to external environments, with their aggressiveness being defined by a combination of temperature, humidity, solar irradiation, and chemically active particles. [3,4]. Since the very beginning of the application of PCMs in various branches of engineering, prediction of their durability at aging has been the focus of attention for numerous researchers, and still remains an important problem [6,7,8,9,10,11,12,13,14,15]. Factors in external environments causing the aging of PCMs are the temperature and humidity of air, precipitation, oxygen, ozone, presence of chemically active compounds, and the ultraviolet (UV) radiation of the sun [5,6,7,8,14,15]. In typical climatic regions (hot dry deserts, humid tropics and subtropics, regions with moderate, low, and extremely low temperatures, etc.), daily and seasonal variations of temperature, humidity, and solar radiation intensity are determined. The data provided in [6,7,8,9] shows a 20–30% and even 40% decrease in strength after a long exposure of PCMs to various climatic conditions. The effect of aging depends on the place of exposure, composition of PCMs, and the measured parameter of interest. As a rule [5,13], the shear strength *τ* and bending strength *σ_b_* are more sensitive to climatic influences than the tensile strength *σ_t_*. For example, carbon plastics are more resistant to aggressive climatic influences than organoplastics. When a PCM is tested under New Zealand and Brazilian conditions, the greatest amount of moisture is absorbed in the specimens [6]. In the conditions of Hawaii, UV radiation is the strongest [6]. The mechanical performance of the PCMs tested in Frankfurt changes to a lesser extent than when exposed in the tropics. It is generally recognized that the atmospheric aggressiveness in temperate climates is lower than in the humid tropics.

Carbon fiber reinforced plastics (CFRPs), glass fiber reinforced plastics (GFRPs), organoplastics (OPs), basalt fiber reinforced plastics (BPs) and other PCMs based on various epoxy, cyanoether, phenolic, and polyester matrices are the most common materials used in construction and other industries [16,17,18,19]. PCM-based structural components during operation are exposed to aggressive environmental factors such as temperature fluctuations, humidity cycling, UV radiation from the sun, precipitation, wind, aggressive aerosols, and mechanical loads. Affected by these factors, materials age and their mechanical properties deteriorate. Extensive data on the effects of PCM aging have been accumulated, including those in [10,13,20,21,22].

An important regularity of the climatic aging of PCMs is the formation of gradients of mechanical parameters across the thickness of the plates. An example is provided in [23], where, after 10 years of exposure in a warm and humid climate, GFRPs based on EDT-10P binder showed the interlaminar shear strength *τ* at the initial values (38 MPa) in the inner layers of an 8 mm-thick plate, but decreased to 21 MPa in the surface layers. After 6 years of similar climatic aging of 5 mm-thick OP Organit 7T plates at a depth of 2.5 mm, the parameter *τ* also retained its original value of 18 ± 2 MPa, while in the surface layer exposed to the sun it decreased to 7–8 MPa.

A similar pattern of change in *τ* was found in CFRP KMU-9TK after 10 year of exposure in a moderately warm climate [24]. Gradients of strength, elastic moduli, coefficient of linear thermal expansion, and glass transition temperature of the polymer matrix over the thickness of the plates form not only in reinforced plastics [23,24] but also in separately-cured epoxy compounds [25] and thermoplastic polymers [26].

The relevance of studies of the inhomogeneity of PCM aging is confirmed by the data of many authors [27,28,29,30,31,32]. For example, it was determined in [27] that in fiberglass based on DEN438 and EPON828, after 2 years of exposure in the tropics, the glass transition temperature Tg of the polymer matrix decreased by 37 °C in the surface layer of the plates, while in the central part of the plates, a similar decrease was only 5° WITH. In [28], a decrease in Tg and an increase in the concentration of amines, ketones, and quinones revealed the effects of the destruction of the epoxy matrix in the surface layer and its absence in the inner layers of fiberglass based on DGEBA.

Determining the gradient of mechanical parameters over the thickness of PCM samples is a complex experimental problem. The works [29,30] show the possibility of revealing the UV degradation profile of a polymer over the thickness of the samples. For this, the strength of thin films cut at different depths from the surface of the polypropylene and polyethylene copolymer samples after UV irradiation in a Xenotest 1200 chamber was measured. It was shown that after 300 h of irradiation, carbonyl groups accumulate in a surface layer 250–300 µm thick. With distance from the surface, the concentration of these groups decreases exponentially. In proportion to this decrease, the strength in the polymer microlayers increases. For polyethylene exposed in a Xenotest 1200 chamber under UV irradiation and in natural conditions of a temperate and subtropical climate, the results are similar to [22]. Relative stretching, density of microtome films, and the content of CH_2_=CH groups determined by IR were found to be sensitive characteristics of the degradation profile over the polyethylene thickness.

According to [31], when epoxy fiberglass was exposed in South Africa, a network of cracks appeared on the surface of the samples, with the structure of the inner layers unchanged. It was established in [32] that the content of C=O and −OH groups increases in the surface layer of an epoxy coating based on DGEBA under the influence of solar radiation.

Thus, under the influence of radiation, temperature, and moisture, the destruction of epoxy matrices occurs on the side of the PCM plates facing the sun. In this layer, the effects of aging are more pronounced [27,28,29,30,31,32].

To clarify the causes of PCM aging the following effects are usually studied:

– plasticization of polymer matrices by atmospheric moisture [33,34];
– swelling (increase in thickness) of samples when adsorbing moisture [35];
– destruction of polymer matrices affected by solar UV radiation and air oxygen [32,36];
– hydrolysis of polymer matrices affected by moisture [37,38,39];
– recoating of polymer matrixes under the influence of temperature and the plasticizing effect of moisture [20,39,40];
– misorientation of organic fibers affected by solar UV radiation, thermal and moisture cycling, and mechanical loads [39];
– structural relaxation and shrinkage of the fibers forming the frame of the reinforcing filler [20,39,40];
– physical aging and structural relaxation of polymer matrices [41,42].

In [43,44] it was shown that the above-mentioned physico-chemical transformations in PCMs lead to microcrack formation on the surface of the samples, in the volume of polymer matrices, and at the polymer–filler interface which reduces the ultimate strength and elastic moduli of the composites.

Thus, the examples considered show that the effects of PCM aging at different depths from the surface are different. The uneven aging is accompanied by the formation of a significant strength gradient in interlaminar compression and shear across the thickness of the plates, and affects the ultimate compressive strength in the central and surface layers of carbon fiber plastics [15,16].

The purpose of this review is to consider the causes of the uneven aging of PCMs under the influence of solar radiation, temperature, and moisture according to the data of dynamic mechanical analysis, linear dilatometry, profilometry, and other methods. Particular attention is paid to the reasons for the formation of microcracks.

## 2. The Role of the Main Factors on the Uneven Aging of PCM

### 2.1. Influence of Diurnal and Seasonal Thermal Cycles

Even in the absence of external mechanical loads, internal stresses occur in PCMs due to differences in the coefficients of the linear thermal expansion (CLTEs) of the components [44,45,46,47]. In the cured composite, a high adhesive bond between the polymer matrix (*m*) and the filler (*f*) results in internal tensile stresses which arise in the matrices $\sigma_{mL}^{T}$ along the fibers *L* and compressive stresses in the fibers $\sigma_{fL}^{T}$ defined by the ratios [44]:

(1) $$\sigma_{mL}^{T}=\;\frac{V_{f}E_{f}E_{m}}{V_{f}E_{f}+V_{m}E_{m}}\left[\left(\alpha_{f}-\alpha_{m}\right)\left(T-T_{0}\right)\right],$$

(2) $$\sigma_{fL}^{T}=-\frac{V_{m}}{V_{f}}\;\sigma_{mL},$$

that depend on the curing temperature *T*_0_, measurement temperature *T*, coefficients of linear thermal expansion *α*, elastic moduli *E*, and volume content *V* of matrices (*m*) and fibers (*f*).

According to [42,43], the levels of internal stresses during daily and seasonal climatic thermal cycles reach up to 40–60 MPa. This is sufficient to form transverse microcracks and decrease the strength properties. As shown in [48], the density of microcracks *D* in CFRP T300/520 increases exponentially with an increase in the number of cycles *N*:

(3) $$\;D=A\left(1-e^{\lambda N}\right),$$

where *A* and *λ* are coefficients depending on the amplitude of thermal cycles. However, thermocyclic internal stresses are equally probable for all monolayers of composites, and cannot cause the observed gradients of mechanical properties across the thickness of aged multilayer plates.

Thus, daily and seasonal thermal cycles are the reasons for the formation of microcracks due to the occurrence of internal stresses resulting from differences between the CLTEs of reinforcing fillers and polymer matrices.

### 2.2. Influence of Moisture

The main cause of non-uniform PCM aging is the action of water [26,49]. In [49], depending on the laying of hybrid multifiber-reinforced thermoplastic polymer composites, after 90 days of exposure to water, the bending strength of the composites [R/R/C/C]s, [R/C/R/C]s, [C/R/C/R]s, and [C/C/R/R]s are reduced by 34.2%, 36.9%, 25.2%, and 25.8%, respectively. In addition, the failure modes of hybrid composites before and after hygrothermal aging vary from brittle failure to ductile failure.

When moisture is absorbed, the linear dimensions of polymer matrices change (swelling) in proportion to the concentration of absorbed water [44]:

(4) $$\epsilon_{m}^{w}=\beta_{m}\left(w-w_{0}\right)=\beta_{m}∆w,$$

where $w_{0}$ is the initial moisture concentration, $\beta_{m}$ is the moisture expansion coefficient of the polymer matrix.

The swelling of polymer matrices creates internal stresses in PCM components; their magnitude is defined by:

(5) $$\sigma_{mL}^{w}=\;\frac{V_{f}E_{f}E_{m}}{V_{f}E_{f}+V_{m}E_{m}}\left(\beta_{f}w_{f}-\beta_{m}w_{m}\right),$$

(6) $$\sigma_{fL}^{w}=-\frac{V_{m}}{V_{f}}\;\sigma_{mL},$$

where $\;\sigma_{L}^{w}$ represents mechanical stresses along the fibers caused by swelling. Calculations [26] showed that hygrothermal stresses peak at the initial moment of moisture diffusion and depend on relative humidity (increase from 25 to 120 MPa).

When swelling with moisture, stresses occur even in separately-cured polymers. For example, when absorbing moisture, an epoxy polymer without a filler showed compressive stresses on the surface of the sample leading to cracking during thermal cycling [50].

Thus, according to the data of [49,50,51], internal hygrothermal stresses are maximum on the surface of PCM plates at the initial moments of moisture sorption and d{esorption. Therefore, the probability of the formation of microcracks in the surface layers increases, which explains the formation of a gradient in the mechanical strength of PCM over the thickness of the samples.

### 2.3. Influence of Oxidation and Physical Aging

Oxidation and physical aging also contribute to the occurrence of stresses and microcracks. This has been shown both for epoxy and bismaleimide polymers and for unidirectional CFRPs based on them [52]. At elevated temperatures, microcracks initiate on the surface of the samples when the critical value of brittleness in the oxidized layer is reached. The cracks then propagate inwards depending on the composition and reinforcement pattern.

In a similar study of an epoxy polymer aged at 150 °C [53], after 900 h of thermal oxidation, a shrinkage layer with a thickness of *ε* = 70 μm was found. Stresses in the polymer layers are defined by the ratio:

(7) $$\sigma =\frac{E\varepsilon }{1-\nu^{2}},$$

where *E* is the modulus of elasticity, *ν* is the Poisson’s ratio ranged from 85 MPa on the surface to −10 MPa at a depth of 200 µm from the surface and caused microcracks.

In [54], the dependence *γ* = *E/E*_0_ − 1 was investigated during thermal oxidation of the epoxy polymer PR520 at 120 °C, where *E*_0_ is the modulus of elasticity in the initial state, and *E* is the modulus of elasticity after aging. As the surface is approached and the duration of the test is increased, the *E* value of the surface layer increases, especially in an oxygen environment, leading to an increase in the brittleness of the surface layer. Compared to the initial bending of 3% (using 4-point bending), the aged specimens failure occurs at 1% bending.

Due to formation of the Young’s modulus gradient, the strength of the epoxide decreases to a greater extent in an oxygen environment, but approaches a certain limit value upon achievement of the highest microcrack density, as follows from relation (3).

Toscano et al. [55] measured the stresses of the epoxy polymers DGEBA and DGEBF, which were moistened and re-dried at 50 and 80 °C. The stress level on the surface of the samples during sorption was −12 MPa and 12 MPa during drying. Harper and Weitsman [56] also showed that drying of moisture-saturated CFRP is accompanied by tensile stresses which can exceed the tensile strength of the material and cause microcracks in the matrix and destruction of the interface. Cracks and delaminations create new surfaces for moisture absorption during subsequent thermal and moisture cycles. It is noted that cyclic exposure (wetting-drying) is more aggressive for CFRP compared to continuous wetting.

The results of [52,53,54,55,56] suggest that the progressive oxidation of PCM polymer matrices in open climatic conditions is one of the probable causes of the formation of mechanical strength gradients across the thickness of the plates.

### 2.4. Combined Influence of Environmental Factors with the Ultraviolet Component of Solar Radiation

If thermal and moisture exposure is accompanied by UV irradiation, its simultaneous or alternate exposure significantly increases the likelihood of microcrack formation. This regularity was confirmed in [57,58,59,60,61,62,63].

When exposed to UV, cracks appeared on the surface of the epoxy polymer SC-15 due to increased brittleness resulting from cross-linking [57]. With the formation of microcracks in the polymer, the compressive strength decreases. After 15 days of UV irradiation, the strength decreases by 22% (from 273 to 212 MPa), the elastic modulus by 16%. After cycles of UV and condensation of water vapor, the strength decreases by 30% (from 273 to 190 MPa), the modulus of elasticity by 27%.

Specimens of CFRP IM7/997 with dimensions 152 × 12.7 × 1.27 mm with laying [0]_8_, [90]_8_ [0/90]_2s_ were tested for tension after aging under the following conditions: UV irradiation in the wavelength range of 295–365 nm with a power of 0.68 W/m^2^ at 60 °C and in an atmosphere of saturated water vapor at 50 °C [58]. CFRP specimens after 500 h of UV irradiation lost 0.27% by weight (volatiles and dissolved moisture). After exposure to a humid environment, the mass of the samples increased by 0.89%. Humidification after UV irradiation showed a similar result (−0.25% after 500 h of UV and +0.8% after humidification. Cycling (UV humidity) resulted in a slight initial increase and a weight loss of up to 1.2% after 1000 h of testing. The combination of UV and moisture produced synergistic effects of extensive matrix erosion, matrix microcracking, matrix-fiber boundary violation, fiber loss, and voiding. For specimens cut in the transverse direction, each type of aging causes a decrease in strength, but the maximum loss of strength was 29% after cyclic exposure to UV and moisture.

Similar effects were found for CFRP IM7/997 [59], GFRP based on epoxy and polyester matrices [60,61], CFRP AS4/8552 [62], and CFRP based on T700 fiber [63]. In all cases, a decrease in mechanical properties of PCM is accompanied by damage at the polymer-filler interface and microcracks.

Thus, under the influence of UV irradiation, chemical reactions of destruction, oxidation, and cross-linking of the polymer binder [64] occur on the PCM surface, enhancing changes in color and gloss, liming, chipping, microcracking, blistering, removal of the polymer resin from the surface without exposing the fibers, complete exposure and delamination of fibers, and delamination of the surface layer. These chemical and morphological transformations initiate the growth of thermomechanical stresses and, as a consequence, formation of cracks on the irradiated surface. In the inner layers of the plates, the density of cracks and the loss of strength decrease, which explains the effects of inhomogeneous aging of PCMs.

A convincing illustration of the non-uniform climatic aging of PCMs is a comparison of the thermal expansion of the surface and inner layers of exposed plates. The classical theory [65] allows expressing the CLTE of a composite along the fiber direction *α_L_* in terms of the corresponding values of the polymer matrix and filler by the formula:

(8) $$\alpha_{L}=\frac{E_{m}\alpha_{mL}V_{m}+E_{f}a_{fL}V_{f}}{E_{m}V_{m}+E_{f}V_{f}},$$

where *E_m_*, *E_f_* are elastic moduli, $\alpha_{mL}$, $\alpha_{fL}$ are CLTE, and *V_m_, V_f_* are specific volumes of matrix (*m*) and reinforcing fibers (*f*). It should be taken into account that PCMs are characterized by significant anisotropy; for example, in glass-reinforced plastics, the difference in the values of the coefficient of linear expansion along and across the fibers reaches 5–20%. It has been experimentally and analytically proven that the formation of transverse microcracks in layered PCMs leads to a decrease in the CLTE of polymer binders [66,67].

In the research [24], the temperature dependences of the relative thermal dilatation $\alpha_{L}\;$ of KMU-9TK carbon fiber plastic along the carbon fibers from the surface layers and the central part of the plate were presented. According to the results of the study, the author came to the conclusion that the shrinkage characteristics of carbon fibers are significantly higher at temperatures exceeding the glass transition temperature of the polymer matrix for the surface layer irradiated by the sun, due to a decrease in $\alpha_{mL}$.

**Figure 1:**
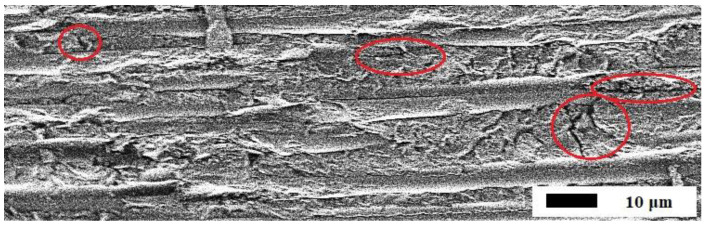
Microstructure of CFRP based on Cycom 977-2 epoxy binder after 6 years of exposure to a warm, humid climate. Typical fractures in the form of microcracks are circled in red. The study of the microstructure of the samples was carried out on a JSM-7800F scanning electron microscope (JEOL, Japan).

Figure 1 demonstrates a typical visual appearance of microcracks formed in PCM 206 during their climatic aging. Typical fractures in the form of microcracks are circled in red. The study of the microstructure of the samples was carried out on a JSM-7800F scanning electron microscope (JEOL, Tokyo, Japan) at a low accelerating voltage in the secondary electron mode. This example shows the microstructure of CFRP based on 207 Cycom 977-2 epoxy binder after 6 years of exposure to a warm, humid climate [68].

Internal stresses cause the appearance of multiple microcracks in the space between carbon fibers, depending on temperature differences (1) and sorbed moisture in the surface layer (5). Based on the results of [68], it can be concluded that daily and seasonal thermal cycles act as an analogue of cyclic mechanical fatigue, cause microcracking with a microcrack density determined by Formula (3) and a decrease in the mechanical properties of PCMs.

### 2.5. Influence of Moisture in a Cold Climate

It should be noted that the main physicochemical transformations listed above are caused by molecularly distributed moisture. According to the data of differential scanning calorimetry (DSC) [69,70], Raman spectroscopy [70,71], X-ray diffraction [72], free water [73], bound freezing water, and bound unfrozen water were revealed in the polymer matrices of PCMs, the share of which is usually predominant. It is this molecularly-distributed moisture that causes plasticization and chemical reactions, the activity of which increases with increasing temperature. Therefore, PCM aging due to the impact of water in regions with warm and hot-humid climates deteriorates the mechanical performance of these materials to a greater extent than after exposure to cold climates [5,6,7,8,9,12].

The effect of cold climates increases for PCMs containing moisture in capillaries, pores, and micro voids. For example, in the porous epoxy polymer studied in [74] by the DSC method, the proportion of freezing water increases with increasing porosity. At low porosity, all water is in a bound state. When temperature decreases, freezing of free and bound water is revealed by an exothermic DSC peak with a heat flux minimum at −18 °C, and when heating, an endothermic peak at 0.6–1.0 °C associated with ice melting is seen. The heat flux absorption peak caused by bound unfrozen water is observed at −38 °C. This result proves that for the transformation of water into ice in the volume of PCMs, depending on the amount of moisture contained and the size of pores, it is necessary to reduce the temperature to −18 °C or more.

According to [69], when the temperature decreases, water concentrated in microvolumes does not crystallize due to the lack of free volume but forms a vitreous state, causing a further increase in the level of internal stresses.

A decrease in temperature by 1 °C increases the pressure by 1.13 MPa. A decrease in air temperature to −60 °C in Yakutsk conditions can increase the internal stresses in PCMs with capillary-condensed moisture to 68 MPa, which exceeds the level of across-the-thickness gradient for a number of PCMs [20] and is another cause of microcracks and the reduction of strength indicators kR.

In [75,76,77,78] the kR indicators of moisture-containing PCMs were studied after cycling in the “cooling-heating” regime. The results of these studies are ambiguous. Therefore, the authors of [75] determined the effect of 20 cycles of 8 h at −18 °C + 16 h in hot water on the properties of the epoxy adhesive bonding plates of water-saturated GFRP. The shear strength of the control specimens was 16.2 MPa and decreased to 9.2 MPa (by 57%) after the cycles. The effect was explained by an uneven distribution of the adhesive and the resulting voids. During freezing, water can generate cleavages due to expansion that weaken the adhesive strength. In [76], a unidirectional 3-layer CFRP based on vinyl ester VE8117 and a separately cured binder were studied at room temperature and 50% humidity. The samples were cured for 25 days in water at room temperature. The water absorption of the polymer samples was 1.3%. The dry and water-filled samples were then incubated at −18 °C or thermocycled with a daily cycle (12 h at −18 °C + 12 h at 20 °C). After 450 days of low-temperature exposure, dry samples increased σt by 9% and water-filled samples decreased this parameter by 14%. After 450 cycles, the σt decreased by 22%. The effect of low temperatures in CFRP is explained by the disruption of adhesive bonding of the fiber with the polymer and the formation of voids.

On the other hand, in [77], the effect of 125 and 250 cycles (−20 to +20 °C) on the adhesion strength of CFRP with steel (adhesive epoxy) was studied. The durability of dry and conditioned samples in distilled and salt water at 45 °C for up to 90 days was measured (water absorption was 1.7 and 1.4%). The effects of the σt and Et reduction characteristics of plasticization were observed in the samples soaked in water. Additional “cooling-heating” cycles showed no significant change in strength. Thermal cycles from −55 to +130 °C, typical for supersonic aircraft flight regimes, were created for IM7/977-2 [78] aviation CFRP. No noticeable difference in mechanical properties was found after 300 cycles for dry and moistened samples with 0.8% water content [78].

Thus, if the water saturation of PCMs does not cause the growth of the defectiveness of the polymer matrix and its interface with fiber, and water is not localized in the form of a separate phase—in microcapillaries—but is a molecularly-distributed polar plasticizer, then additional cycling in the regime “cooling-heating” does not significantly degrade the mechanical properties of PCMs.

If water is localized in the macrodamages of PCM (cracks) in free or capillary condensed states, its transformation into ice at temperature decrease promotes the additional growth of internal stresses; indicators of arising microdamages in a polymer matrix and on the border of its interface with a filler are the rms stress of acoustic emission.

This conclusion is confirmed by the model experiment in the work [79]. The effect of low temperature on the properties of dry and water-saturated fiberglass KAST-V was studied by the method of acoustic emission (AE). KAST-V specimens cut from 2.5 mm-thick plates had dimensions of 130 × 30 mm. For a part of the specimens from the side of the edge, splitting was carried out with the formation of a crack, located between the layers of the composite. The specimens were dried in the thermostat and then subjected to water saturation. After that, the specimens were cooled to the temperature of dry ice with the recording of acoustic emission during the cooling process according to the method [79]. RMS acoustic emission voltage U was used as an informative parameter. AE signals were recorded in the frequency range from 50 to 500 kHz. The signals were recorded with a sampling frequency of 2.5 MHz.

**Figure 2:**
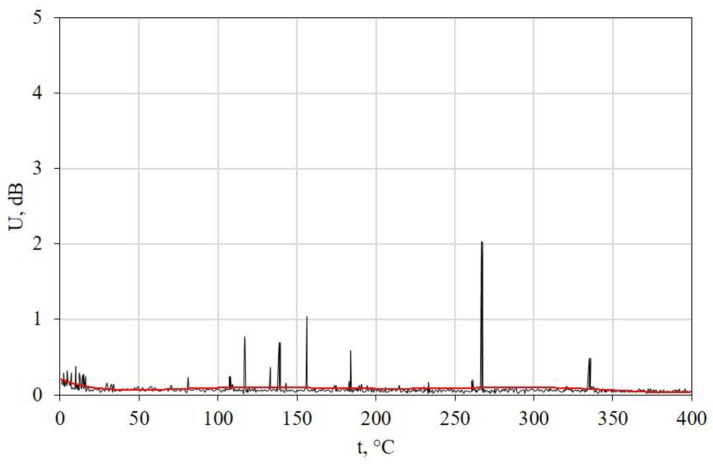
RMS AE stress of the dried sample of KAST-V with a 15.7 mm crack length [79].

The measurements showed that for all dried specimens, as well as for water-saturated specimens with surface defects during cooling, the AE is within the background noise limits. The characteristic example for a dried specimen with a 15.7 mm long crack is shown in Figure 2. The AE parameter U is characterized by small single emissions caused by mechanical noise from the movement of dry ice pellets after putting the specimen into the container.

**Figure 3:**
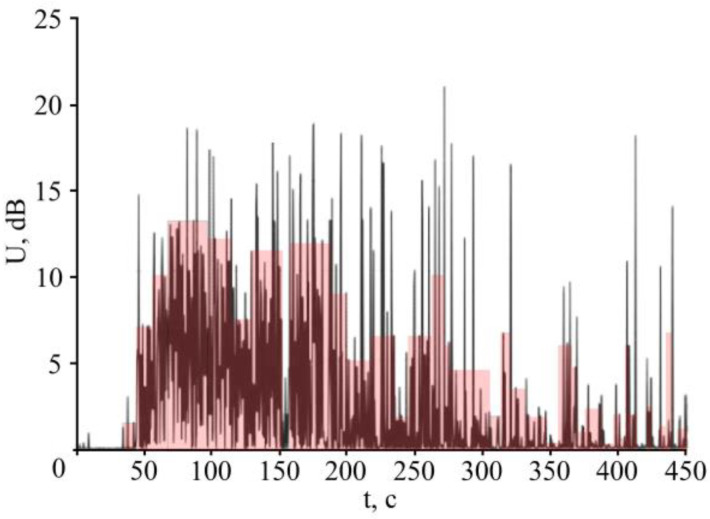
RMS AE stress of the dried KAST-V specimen with a 15.7 mm crack length [79].

The AE for KAST-V specimens containing 0.98% water with edge-split layers is different. As can be seen from Figure 3, acoustic emission with the pulse amplitude exceeding the background by more than two orders of magnitude is observed in these specimens. This allows us to relate the AE pulses to the processes of ice crystallization, the increase of internal stresses during the transition of water into ice at the crack tip, and multiple acts of microdamage of the binder, which generate acoustic pulses.

The examined results show that the gradients of mechanical parameters over the thickness of aged PCMs are caused by the dominant damage to the surface layers of the plates due to the propagation of microcracks under the influence of thermal and moisture cycling enhanced by exposure to solar UV irradiation. To model non-uniform aging, additional information is needed on the density of microcracks across the thickness of the plates for various types of PCM climatic tests.

The analysis carried out showed that under the influence of UV components of solar radiation, even in a cold climate, the surface of PCMs undergoes destruction and microcracking, increasing the number of sources of internal stresses. Seasonal and daily thermocycles worsen mechanical properties of composites, especially if freezing water accumulates in their micropores and capillaries. Examining this pattern, we can consider modeling the aging of PCM in cold climates.

## 3. Modeling Uneven Aging of PCMs

Predicting the change in the strength of PCMs during uneven aging is still a difficult problem. Satisfactory solutions have been obtained only for certain simple cases. In [49], the model for the evolution of moisture diffusion and behavior during hygro-thermal aging was constructed using finite element analysis. At the same time, a clear convergence with the experimental results of moisture absorption was obtained and differences in the evolution of moisture diffusion in R/C hybrid composites were shown. In [80], the thermal moisture aging of glass-reinforced plastics on a VK-36r binder was studied. This material was kept for 150 days at a temperature of 90 °C and a relative humidity of 98 ± 2%. During aging, a linear decrease in compressive strength (σ_c_) was found with an increase in the moisture content (w) to 3.9%. To simulate aging, the formation of microdefects ν under the action of water is assumed. In the process of moisture saturation, defects move into the inner layers of fiberglass according to Fick’s law

(9) $$\frac{\partial \nu }{\partial t}=D_{h}\frac{\partial^{2}\nu }{\partial h^{2}},$$

where *ν* is the concentration of defects at depth h from the surface at time *t*, *D_h_* is the diffusion coefficient of defects, which coincides with the diffusion coefficient of moisture to the depth of the samples. The diffusion model of defects (9) satisfactorily describes the experimental dependence of σc on the aging time.

The theoretical approach to modeling the non-uniform aging of PCMs is considered in [81]. It was assumed that damage occurs during aging, such as microcracks and embrittlement of the polymer matrix of the material. Damage moves linearly into the inner layers of the plate with unidirectional reinforcement. In this case, the influence of moisture sorption and desorption is not taken into account, and the properties of the reinforcing fibers are considered unchanged. The generalized Hooke’s law is presented in the form

(10) $$\sigma_{ij}=C_{ijkl}^{0}\varepsilon_{kl}+N_{ijkl}\varepsilon_{kl},$$

where $\sigma_{ij}$ is stress tensor, $\varepsilon_{kl}\;$ is strain tensor. The first term includes the stiffness tensor $C_{ijkl}^{0}$ for a volume element without defects. In the second term, $N_{ijkl}$ is the stiffness tensor, which takes into account the shape of defects.

The calculation of the tensor $N_{ijkl}$ in Equation (10) is presented in a general form for ellipsoidal microcracks. For the practical use of the theoretical approach [81] in cases of the uneven aging of PCMs, it is necessary to have additional detailed experimental information about the structure of the composite reinforcement, the shape and size of defects in the damaged layer, the rate of defect propagation, the effect of moisture, the stability of reinforcing fibers, and other factors. Therefore, a systematic study of gradients of strength indicators and physical characteristics of PCMs at different depths of plates from the surface is important for further modeling of uneven aging of PCMs.

In [20], a model based on the assumption of a linear law of damage summation under the action of external factors was proposed to extrapolate the results of full-scale tests of PCMs. This approach involves full-scale and accelerated tests, the results of which reveal the limit states of the studied material (maximum degree of hardening, limit levels of plasticizing action, internal stresses, destruction, etc.) with varying regimes and duration of aging. The possibility of modeling the mechanical parameters *R* by the time dependence is shown:

*R* = *η*(1 − *exp*(−*λt*)) − *β*(1 + *χt*) + *R∞*, (11)

where *η* and *β* are material parameters determined by laboratory accelerated methods; *λ* and *χ* are material and environmental characteristics. The validity of dependence (11) has been tested and confirmed for different sets of experimental data obtained during exposure in different climatic zones.

Prediction of conservation indices (*k_R_*) by means of rigorous physical models is apparently still impossible due to a large number of significant influencing factors and the insufficiently-studied synergistic effects of seasonal and diurnal temperature, humidity, and solar radiation variations. In our opinion, extrapolation methods can be used to predict the mechanical properties of PCMs.

## 4. Conclusions

Long-term exposure of PCMs in open climatic conditions causes an irreversible change in their mechanical and various physical parameters due to plasticization, swelling, hydrolysis, post-curing, and the destruction of polymer matrices under the influence of temperature, humidity, and UV radiation from the sun. An important regularity of the climatic aging of PCMs is the formation of gradients of mechanical parameters across the thickness of the plates. An example is provided in [23] where, after 10 years of exposure in a warm, humid climate, GFRP based on EDT-10P binder showed the interlaminar shear strength τ at the initial values (38 MPa) in the inner layers of an 8 mm-thick plate, but decreased to 21 MPa in the surface layers. After 6 years of similar climatic aging of 5 mm-thick OP Organit 7T plates at a depth of 2.5 mm, the parameter τ also retained its original value of 18 ± 2 MPa, while in the surface layer exposed to the sun, it decreased to 7–8 MPa. A similar pattern of change in τ was found in CFRP KMU-9TK after 10 years of exposure in a moderately warm climate [24].

The occurrence of gradients of indicators must be taken into account when modeling the strength of PCMs for long periods of operation. The progress in modeling the strength of PCMs under long-term exposure in open climatic conditions depends on obtaining new experimental information about the microdamage profiles along the PCM thickness associated with the dominant impact factors.

The present review has analyzed the known causes of the uneven aging of carbon fiber reinforced plastics, fiberglass, and other PCMs which are used in construction and various branches of engineering under the influence of solar radiation, temperature, and moisture. As the result, it is possible to draw the following conclusions:

1. The dominant cause of the uneven aging of PCMs is the effect of water. When swelling with moisture, internal hygrothermal stresses arise which are distributed unevenly over the thickness of the plates. The maximum internal stresses are maximum on the surface of the PCM plates at the initial moments of moisture sorption and desorption. Therefore, the probability of the formation of microcracks in the surface layers increases. The unevenness of aging depends on changes in air temperature and humidity, and increases with daily and seasonal transitions from sorption to desorption.
2. Progressive oxidation of PCM polymer matrices under open climatic conditions in combination with the action of UV radiation is one of the probable reasons for the formation of mechanical strength gradients across the thickness of the plates.
3. Physical and chemical transformations in PCM polymer matrices activated by temperature, moisture, and solar radiation promote the capillary condensation of moisture, which can turn into a solid phase at temperatures below 0 °C, be a source of additional internal stresses that cause the formation of new damage in the surface layers, and be the reason for the decrease in the strength of PCMs.

Predicting the change in the strength of PCMs during uneven aging is still a difficult problem. Satisfactory solutions have been obtained only for certain simple cases. An urgent research task for the coming years is a comprehensive microscopic study of the density and shape of microcracks at different depths from the surface, depending on the composition and structure of the PCM reinforcement and the current climatic factors.

## Data Availability

The data used to support the findings of this study are available from the corresponding author upon request.
